# Understanding Perceptions of Climate Change, Priorities, and Decision-Making among Municipalities in Lima, Peru to Better Inform Adaptation and Mitigation Planning

**DOI:** 10.1371/journal.pone.0147201

**Published:** 2016-01-25

**Authors:** Mariella Siña, Rachel C. Wood, Enrique Saldarriaga, Joshua Lawler, Joseph Zunt, Patricia Garcia, César Cárcamo

**Affiliations:** 1 School of Public Health and Administration, Universidad Peruana Cayetano Heredia, Lima, Peru; 2 Department of Global Health, University of Washington, Seattle, Washington, United States of America; 3 School of Environmental and Forest Sciences, University of Washington, Seattle, Washington, United States of America; Queensland University of Technology, AUSTRALIA

## Abstract

Climate change poses multiple risks to the population of Lima, the largest city and capital of Peru, located on the Pacific coast in a desert ecosystem. These risks include increased water scarcity, increased heat, and the introduction and emergence of vector-borne and other climate sensitive diseases. To respond to these threats, it is necessary for the government, at every level, to adopt more mitigation and adaptation strategies. Here, focus groups were conducted with representatives from five Lima municipalities to determine priorities, perception of climate change, and decision-making processes for implementing projects within each municipality. These factors can affect the ability and desire of a community to implement climate change adaptation and mitigation strategies. The results show that climate change and other environmental factors are of relatively low priority, whereas public safety and water and sanitation services are of highest concern. Perhaps most importantly, climate change is not well understood among the municipalities. Participants had trouble distinguishing climate change from other environmental issues and did not fully understand its causes and effects. Greater understanding of what climate change is and why it is important is necessary for it to become a priority for the municipalities. Different aspects of increased climate change awareness seem to be connected to having experienced extreme weather events, whether related or not to climate change, and to higher socioeconomic status.

## Introduction

The risks associated with climate change are increasing worldwide. Some effects of climate change are already impacting populations, and those impacts are projected to worsen and multiply in the future [1]. The global effects of climate change are varied, and include altered weather and precipitation patterns, increased rates of glacial melt [2], rising temperatures [3], changes in extreme temperatures, ocean acidification [4], and sea-level rise [5]. These can affect human health directly through extreme weather such as floods [6] and increases in the incidence of heat-related illnesses [7], and indirectly through changes in disease vectors and their related habitats [8,9], reductions in the quality and quantity of water [10], and decreases in agricultural production [11] that result in nutritional deficiencies [12].

Lima is Peru’s most populous city with more than 9 million residents—one third of the country’s total population [13]. Located in a desert ecosystem with little rain; the population is dependent on rivers flowing from the Andes, which are largely fed by glacial melt, for water for consumption and domestic uses. By 2030, in Lima, precipitation could be reduced by an estimated 20–30% near the coast, and up to 10% farther inland. Also, temperatures are projected to increase throughout the country by approximately 0.4°C by 2030 [14]. Glacial melt in the Andes due to increasing temperatures and changes in rainfall could make the supply of water less reliable and more expensive [15]. Diminished water will potentially generate problems for agriculture, hydroelectricity generation, and subsistence livelihoods, unless adequate adaptation measures are taken.

Adopting strategies in Lima to both adapt to and mitigate climate change is necessary to protect public health, and can be economically advantageous in the long run [16]. It is often the responsibility of local and regional governments to adopt and implement these strategies, especially those that require government funding and resources, long-term development, and support [17]. The municipal governments of Lima could serve a key role in leading the response to climate change. Thus, it is critical for the municipalities to understand the consequences of climate change for the population, and to implement adaptation and mitigation strategies to ensure preparedness in the face of increasing climate change impacts.

Other studies have analyzed perceptions of climate change across individuals in various locations and with different occupations [18–21]. Fewer have assessed the perceptions of climate change at the municipal level, as opposed to the individual, to determine how it is viewed and addressed within governing bodies as a whole [22]. In this study, the view of the entire municipality was evaluated by holding focus groups with municipal officials that hold leadership roles and have the potential to advocate for increased adoption of climate change adaptation and mitigation measures. Understanding the municipal decision-making process can provide insight into possible incentives for action, and ultimately, health and social benefits.

Five municipal districts in eastern Lima, Peru were selected to participate in focus groups, and municipal officials were asked about their knowledge and awareness of climate change, the level of importance climate change receives within their municipality, the decision-making process regarding the allocation of funds to implement various public projects and initiatives, and their priorities. An emphasis was placed on determining the gaps between 1) understanding of climate change and other environmental and public health related issues and 2) steps toward addressing these issues.

## Methods

### Study Design

This qualitative study applies the principles of phenomenology to understand the perceptions of climate change within several municipalities of Lima. Focus groups were used to collect data on how the municipalities as a whole view climate change and how it applies to their work.

### Study Site

Five municipalities of eastern Lima, Peru were selected to take part in focus groups. East Lima comprises a majority of the population of Lima, encompassing some of the largest and most diverse districts. This region is also likely to be more vulnerable to climate change. Due to their proximity to the river and the mountains, the people in this region have been historically subject to more frequent landslides and flooding.

To provide a representative sample, these municipalities were selected based on their diversity of geography, population, capacity, and SES (socioeconomic status) index level (Table 1). SES is defined and calculated by the Asociación Peruana de Empresas de Investigación de Mercados (Peruvian Association of Market Research Companies) and is based on: housing tenure; predominant construction material of walls, roof, and ceiling; household appliances; access to health services; the head of household’s education level and job; and monthly family income [23].

**Table 1:** Relevant study information and characteristics of each municipality*.

Characteristic	M1^+^	M2^+^	M3^+^	M4^+^	M5^+^
Focus Group Date	24 Nov 2014	25 Nov 2014	28 Nov 2014	16 Dec 2014	9 April 2015
Population	190 961	212987	611082	166912	1069566
Area (km^2^)	12.5	236.5	77.7	65.8	131.3
Number of homes	46 872	48122	134002	39121	240938
Density (citizens/km^2^)	15276.88	900.58	7864.63	2536.66	8145.97
Predominant SES**	38.9% (D)	47.3% (C)	38.6% (C)	44.1% (A)	56.6% (D)

*Source: Ipsos Perú [24,25].

^+^The names of the municipalities are concealed. The denomination M1 through M5 was assigned based on the order in which the focus group took place.

**The SES levels are A, B, C, D, and E, with A being the highest and E the lowest.

### Data Collection

In each of the five municipalities (hereafter referred to as M1 through M5), hour-long focus groups were held with municipal officials representing various departments of the municipality. The date of each focus group is detailed in Table 1. The same protocol was followed for each municipality, and included contacting the municipality, sending a letter of invitation to the Mayor’s office with a list of desired participants, following up with the Mayor’s office to ensure permission for the focus groups and distribution of the invitation, coordinating date and time of the focus groups, and executing the focus groups with the aid of a trained facilitator. Each focus group was conducted by the same facilitator who has extensive experience in qualitative research as well as in climate change and public health topics. Participants were identified through the use of publicly available information on the municipality’s website, and their attendance was subject to their availability. The number of participants varied between 5 and 12 in each focus group. Focus groups were held in a meeting room at the municipality headquarters and only the participants, facilitator, and researchers were present at each focus group.

The municipal departments from which representatives were invited were chosen to be diverse enough to present a comprehensive viewpoint, yet narrow enough to increase the likelihood that they would be interested in current and potential impacts of climate change or other environmental issues. Each department was selected based on the possibility that their work could be impacted by climate change, or the municipal official was in a position that may include approving or managing projects with the capacity to mitigate or adapt to climate change. The departments represented in the focus groups varied slightly between municipalities but largely included public works, health, environment, planning, public investment, education, culture, and transportation.

The participants were asked open-ended questions from a semi-structured guide (S1 Appendix) on topics including the municipality’s priorities, views on the importance of human health and the environment, perception of climate change, effects of climate change within the municipality, activities that cause climate change within the municipality, and their decision-making process for adoption of public projects. They were also asked about any sort of tools they use in their decision-making process, such as rating systems or cost-benefits analyses, and whether a tool that incorporated environmental and health considerations to assess climate change adaptation and mitigation strategies would be welcome and useful. Audio recordings were made of each session.

The participants were not informed beforehand which issues would be discussed at the focus group so they would be more likely to give their true opinions, rather than what they think would be the more desirable answer, and thus avoid social desirability bias [26]. When the potential participants were invited to the focus groups, they were given the names and host-institution of the researchers to ensure confidence, and were told that the topic of the focus group would be the municipality’s decision-making process and priorities for investment in projects. The focus groups were structured so that the municipality’s priorities and the variables and processes of project implementation were covered first, followed by questions regarding priorities specifically related to human health and the environment. Climate change was brought up last, with questions covering awareness and perceived causes and effects of climate change within the municipality.

### Data Analysis

The recordings of the focus group sessions were transcribed in full. Three of the investigators read the transcripts to establish an individual group of themes and sub-themes. The investigators then met to discuss and agree upon a final set of themes and sub-themes (codes), with corresponding definitions. These final codes were applied to each transcript and were then analyzed to determine the primary topics across all the focus groups, and to compare similarities and differences. All coding was completed using Dedoose Version 5.0.11 (Los Angeles, CA: SocioCultural Research Consultants, LLC. 2014) software. Transcripts were not returned to participants for comments or corrections.

### Ethics

This study was approved by the Institutional Ethics Committee of the Universidad Peruana Cayetano Heredia (reference number 63551). Written informed consent was collected from all participants prior to their participation in the study.

## Results

### Municipal priorities

**Fig 1:**
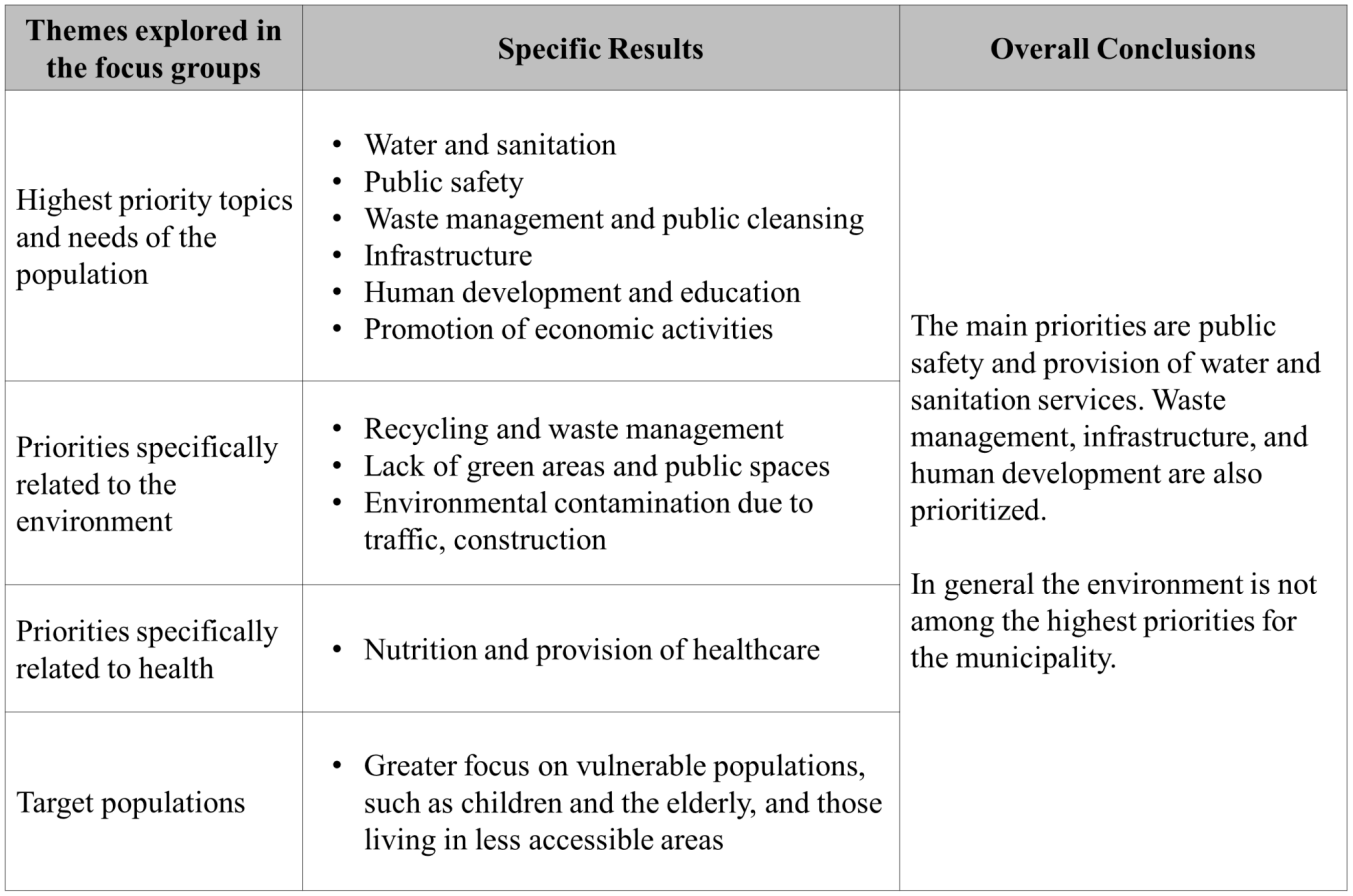
Assessment of priorities and needs that receive the most attention and focus.

The main priorities in the municipalities revolved around issues related to public safety and provision of essential services like water and sanitation (see Fig 1).

There was no mention of the environment as a priority except in M4, which was the only municipality to mention climate change and projects related to the environment before the facilitator brought up those subjects. M4 also showed a greater focus on human development, displaying an interest in projects with more long-term benefits. This difference could be attributed to the fact that they have a higher socioeconomic status (SES). M4 acknowledged this, as demonstrated in the following quote.

*Because of us having only 25% of the district without access to basic public utilities*, *I think we can afford to implement human development projects as public investment projects*.

This shows a greater level of foresight and planning for the future that is consistent with their greater awareness of climate change. However, the representatives from M4 also reported that they previously had to overcome a lack of support and approval from higher government levels for these public investment projects.

*It was highly criticized by the Ministry of Economy*, *to do public investment projects of a social character*. *They thought that that was a way to avoid taxation*.

### Perceptions of climate change

**Fig 2:**
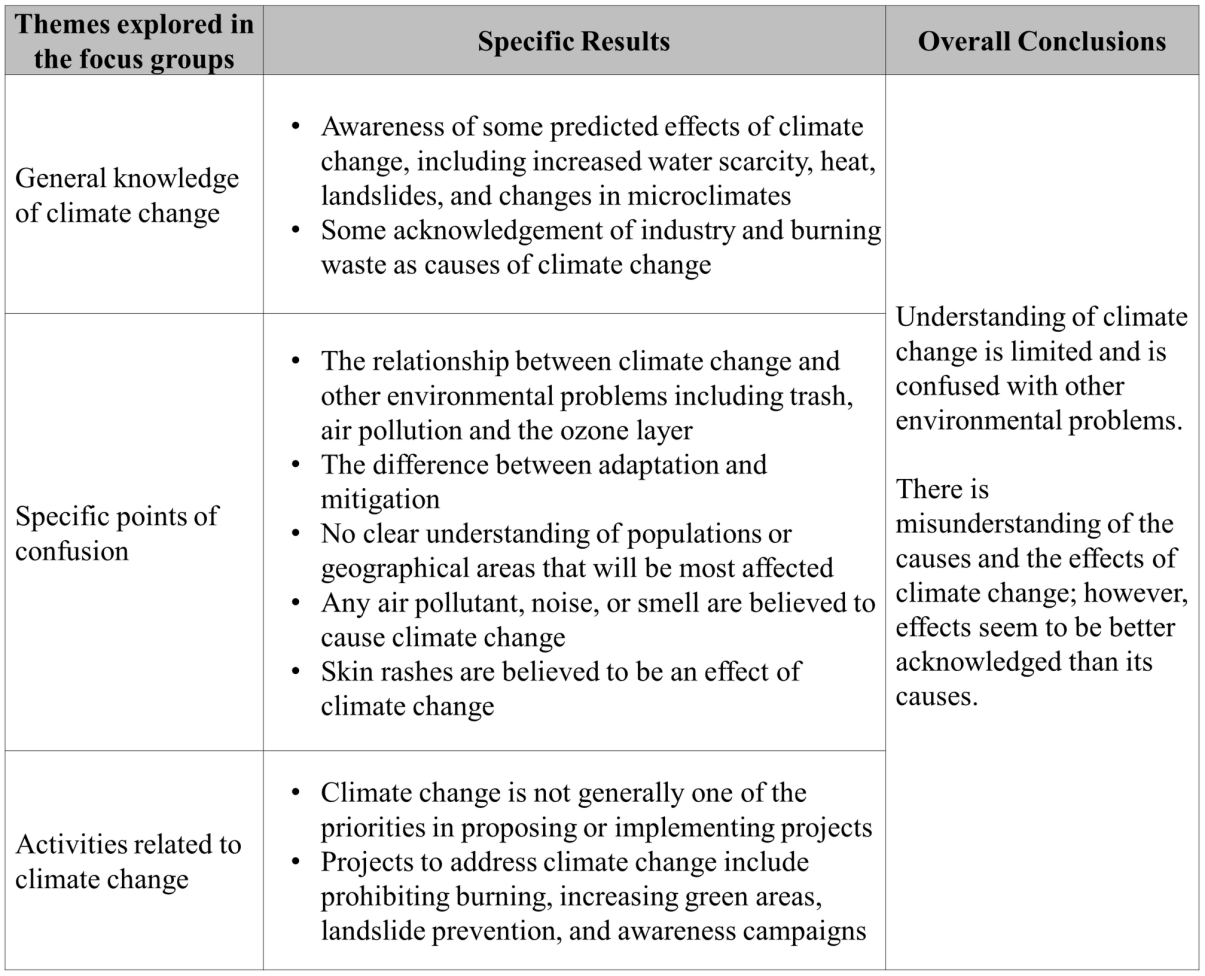
Determination of the perceptions and levels of awareness of climate change.

Understanding of climate change, and its causes and effects, is generally limited and is often confused with other environmental problems such as air pollution, solid waste, and the ozone layer (see Fig 2).

Such confusion can be seen in the following two quotes from M3, where other environmental issues were mentioned when they were asked directly about climate change.

*Facilitator*: *And here*, *in the municipality*, *is there concern about climate change*?

*Participant*: *Just today we had a meeting with the Health Ministry*, *with the Environmental Health Department*. *We are organizing a campaign by the end of December*, *about recycling*, *nutrition*, *and also to protect the ozone layer*.

*Facilitator*: *Regarding strategies*, *for climate change*, *here in the district*, *do you take certain actions*?

*Participant*: *For example raising awareness campaigns*, *for the population*, *about recycling*.

The participants from M4 showed the least confusion about climate change; of all the municipalities, they were the most knowledgeable about the causes and effects of climate change, and their information was the most accurate.

Despite any confusion, all the municipalities showed willingness to plan activities and projects to raise awareness and take action. In the case of M2, some are already implementing campaigns to raise awareness of climate change in their population. M2 described an example in the following quote.

*We*, *as the Public Cleanliness subdepartment*, *we are also linked closely to the Climate Change issue*, *we have fifteen environmental promoters*, *that educate*, *for example*, *on the subject of climate change*, *they teach not to burn waste*, *not to burn their waste because that generates gases*.

The awareness of climate change amongst the representatives from M2 may stem from the fact that they have experienced natural disasters in the form of landslides, which they attribute to climate change. This experience appeared to have some bearing on their perception of climate change.

*I think that landslides would be the main problem [associated with climate change] that could happen along the years*, *not now*, *but over the years*, *if there is such a strong temperature variation that could be noticed here in [municipality] and could make us subject to a streak of landslides*.

M2 perceives landslides to be associated with climate change, and due to their geography, they have a greater risk for landslides than the other municipalities included in this study [27]. Whether or not the link between landslides and climate change actually exists, the perceived association still contributed to a heighted sense of awareness of issues related to climate, and a better understanding of the importance of actions to address climate issues.

The importance of water resources and their relation with climate change was one of the more widely known effects recognized by all the municipalities, as is exemplified by the following quote from M3.

*Taking care of the water is very important*, *because now with the climate change issue pretty soon we will not have water*.

Geographical, socio-economical, and technological factors that would increase vulnerability to the effects of water scarcity are also recognized in all municipalities, as is shown in the following two quotes from M5 and M4, respectively.

*Lima is a desert zone*, *right*?…*It is totally dry*, *therefore we have to transfer*, *transport water from the river*, *from the wells that are close to [remote location]*. *And to bring it is very expensive*.

*Practically 70% of green areas are irrigated with drinking water*. *I was mentioning the issue with potable water*, *which is needed in other places*, *because here we can see that we are living in a desert*, *it is a tremendous situation*. *…Now*, *the neighbor wants green areas*, *first problem*: *no water*.

Both municipalities also expressed their intent to construct water treatment plants that would generate water to irrigate green spaces. M5 also encompasses areas that are particularly susceptible to landslides, and during the focus group with them, it was noted that recent landslides in another district had greatly influenced their awareness of climate change and its effects. The following quote shows how this event affected the perception of climate change in M5.

*Facilitator*: *Have you noticed if there is some concern about climate change*?

*Participant 1*: *Yes*, *what is happening now for example*, *we are in a situation that could cause a problem*, *which is the Rimac river*. *We*, *as a prevention measure*, *we are working on cleaning it [to avoid flooding due to involuntarily damming the river with rubble]*. *…Because you know that the rivers can in any moment make of the [main] avenue its riverbed*, *so we have to be prepared*. *…Another thing that worries us a lot about the subject of climate change is that this year El Niño is announced; El Niño brings consequences such as heavy rain*. *Many settlements on the hills don`t have retaining walls and they are on cliffs*. *After the last El Niño*, *we already know that with the rain usually these cliffs glide*, *causing the houses to sink*, *so that subject is also priority*.

*Participant 2*: *Previously had happened something similar to [recent landslide disaster place]*, *two years ago*, *it has rained too much in the [hills] and it has split houses*.*…*, *so climate change is a serious problem that we have to prevent now*.

The recent landslides appear to have contributed not only to an increased awareness of climate change, but also to an increased sense of urgency. However, this quote also shows confusion between anthropogenic climate change and natural climactic cycles, such as El Niño.

### Decision-making processes

**Fig 3:**
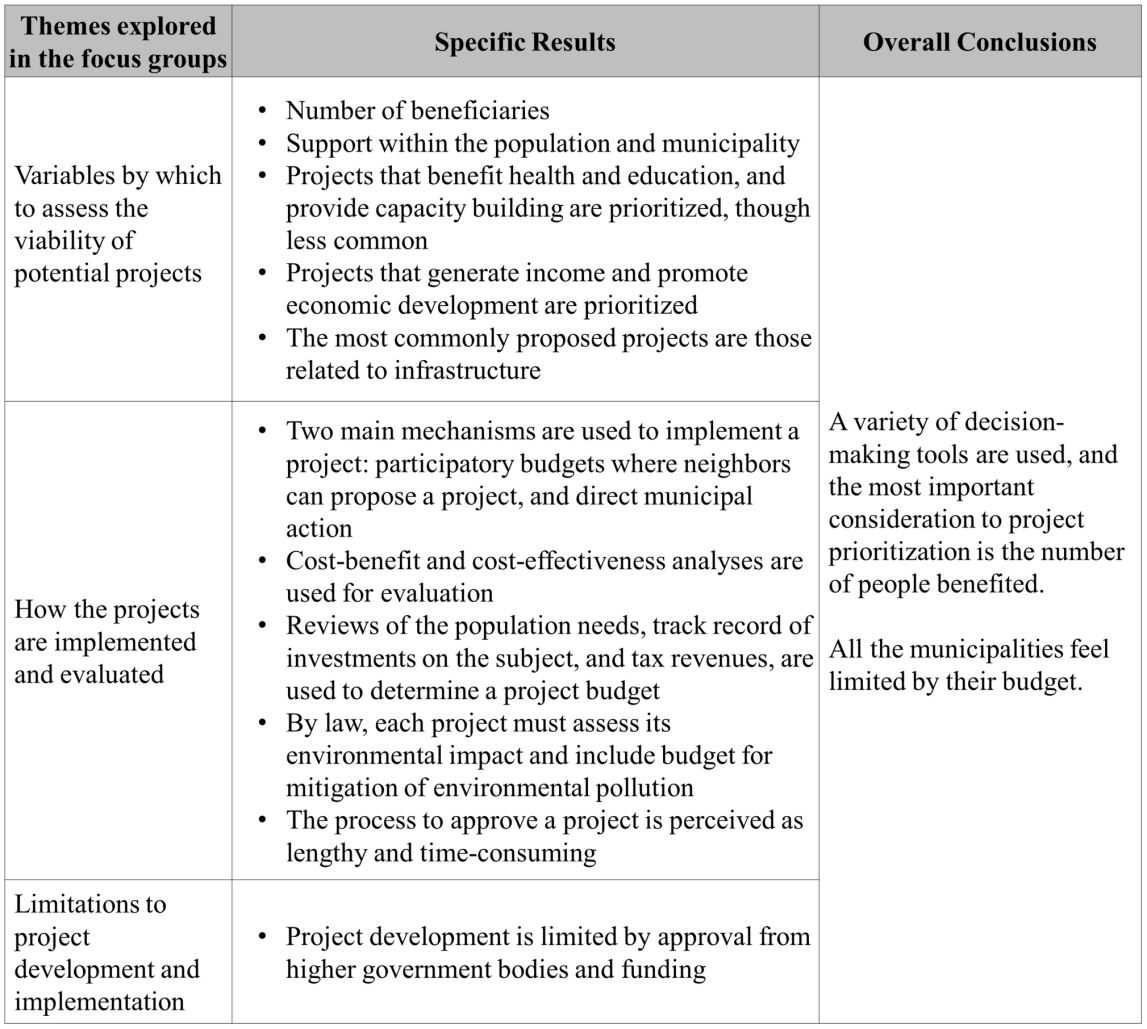
The decision-making process and how the municipal budget is allocated.

There was an emphasis on economic development in many of the municipalities’ decision-making processes (Fig 3).

A quote from M1 accurately represents how the municipalities think of prioritizing projects, and the reasons behind why they are prioritized.

*Boulevards are made with the aim to generate jobs*. *So that more commercial stores are built within the boulevard*, *not the kind that operate at night*, *but family oriented businesses*.

It shows a focus on projects that are meant to stimulate economic development and job creation, especially in a municipality of a lower SES. There was also a clear focus on human health as a factor with which to evaluate potential projects in all the municipalities. M3′s scoring system reinforces the focus on health, but the general interest seems to be in economic development and on infrastructure in particular, as can be seen in the following statement.

*…We give a higher score to those projects that are related to education*, *health*. *However*, *it is somewhat paradoxical because we assign more points to projects of this kind or nature; however*, *most of the participants from the population don’t propose social projects; they are more oriented to infrastructure projects*.

### Reception of a tool for climate change adaptation and mitigation strategies

When asked, all the municipalities showed at least some level of interest in a tool that would allow them to evaluate and prioritize different climate change adaptation and mitigation strategies, and acknowledged that it could be helpful. They would prefer this type of tool to be interactive, and to provide complete results in a timely manner. Additionally, they would be more likely to use it if it were required.

## Discussion

Knowledge regarding climate change and prioritization of actions to prevent or mitigate the effects of climate change varied across municipalities in Lima. The greatest understanding of climate change and environmental issues was found in M4, which has the highest SES. The municipalities of a lower SES expressed greater interest in immediate, more short-term solutions and issues, particularly those that could be readily seen and felt, such as waste and air pollution. Higher SES is associated with less skepticism and greater awareness of environmental problems and climate change, which is based on the idea that people are more likely to be concerned with issues outside of their basic needs, once those needs have been met [28]. Additionally, as has been previously observed in Lima, environmental management seems to be organized around economic interests reinforced by technocratic arguments [29], which further emphasizes immediate economic needs over long-term planning.

One of the most relevant variables in the calculation of SES is the education level of the head of household [23]. Education has been previously shown to be associated with increased belief in climate change in the United States, though this effect is tempered somewhat by political party affiliation [30]. Likewise, higher levels of education are associated with an elevated awareness of climate change among smallholders in Ethiopia, as well as in several other African countries including Nigeria and Kenya [31]. This study did not collect information on the education of each of the individual participants of the focus groups; therefore, it cannot be implied that education is an explanatory variable for increased climate change awareness in municipalities with a higher SES.

The environment is often perceived as what can be seen, such as animals and landscape, outside of humans and their domain [32]. The intangibility of climate change is sometimes an impediment to acceptance and action [33]. The people’s belief in climate change seems to be largely dependent on their perceived changes in salient weather and experiences with extreme weather [34]. Previous research has shown that groups that have experienced some effects of climate change are more likely to be aware of it and consider it a pressing issue [19]. This could provide some basis for the awareness of climate change in M2, where, despite their confusion regarding their knowledge of certain facts about climate change, the representatives and the municipality recognized the increased risk and had started to implement response programs.

Almost all metropolitan Lima, and especially eastern Lima, is subject to landslides, and based on the climatic variations during El Niño of 1997–98, the province of Lima has been classified as being at medium to high risk for landslides associated with uncommon rain [27]. El Niño events have been linked with increases in rainfall and flood events along the coast of Northern Peru [35], and with temperature increases in Lima [36]. However, the relationship between El Niño events and rainfall in Lima is less clear, where some events have led to increases in rainfall, and decreases in others. Additionally, it has been suggested that flooding in Central Peru is not necessarily associated with excess rainfall [37]. Climate change is projected to cause an increase in surface water temperatures of the equatorial eastern Pacific, which in turn would induce an increased frequency of extreme El Niño events [38]. Climate change predictions for Lima include a decrease in rainfall, showing that the relationship between landslides, extreme weather events such as El Niño, and climate change is complex, though the municipalities tend to think of them as linked.

The observed confusion between climate change and other environmental problems is not uncommon, and has been reported elsewhere. A study of health professionals in China showed that respondents mistakenly believed that chlorofluorocarbons (CFCs) were among the main greenhouse gases [39].

From December 1 to 12, 2014, Lima hosted the Twentieth Conference of Parties (COP20), a summit on climate change that brought together key decision-makers and representatives from over 190 countries of the United Nations Framework Convention on Climate Change (UNFCCC) to share information and ideas, and draft an agreement on steps to manage and fund efforts to reduce the increasing effects of climate change [40]. The focus groups with M1, M2, and M3 were held in the week preceding the start of the COP20, while the focus groups with M4 and M5 were held after the COP20. The focus group with M4 was in the week following the COP20, and the focus group with M5 was delayed by 4 months. This may have contributed to a higher level of awareness and sense of gravity regarding climate change in M4 and M5. The COP20 received a significant amount of media coverage, and precipitated many different side events and related activities in the months and weeks before the official start [41]. Given this information, it is possible that heightened awareness of climate change was brought about by the COP20.

Another instance where timing of the focus group could have affected the municipality’s understanding of climate change is in the case of M5, where the focus group was held in the weeks following landslides in another part of the city. Their occurrence raised awareness for possible causes, such as increased precipitation and climatic changes [42]. While the municipality certainly could have had some level of understanding and concern over climate change without this event, it appears to have brought the issue to the forefront.

What remains to be seen is whether heightened awareness attributed to either the COP20 or landslide will be sustained. It has been previously suggested that media coverage of climate change in Peru is event-driven. An increase in coverage from 2007 to 2008 coincided with the COP13 meeting in Bali, release of the 2007 IPCC reports, and the Latin America and European Union summit, which was held in Peru in 2008 and focused on poverty and climate change. However, in 2009 and 2010, coverage declined [43], and it is unclear whether climate change will continue to be a pressing issue after the media attention related to the COP20 and the landslide has subsided. This study is limited in that the perceptions gained from the municipalities represent their thoughts and interests at a single time point, rather than over time.

## Conclusions

The results of this study provide a basis for understanding how climate change is perceived among governing bodies of Lima, and provide information on priorities of the municipalities, and their decision-making process regarding public programs and initiatives. Climate change and other environmental issues were not prioritized, although they were generally recognized as being important to the health and safety of the population. Confusion surrounding climate change, and its causes and effects, may be a factor in why it was not a priority and often not considered when deciding which projects to pursue.

Strategies to adapt to and mitigate climate change could have long-term benefits for the population of Lima, in terms of both health and economics. Better understanding of climate change and the strategies for mitigation and adaptation could be achieved through development of a tool to help municipal officials evaluate different strategies.

## Supporting Information

S1 Appendix Focus Group Questionnaire—Facilitator’s Guide. (DOCX)
