# Supplementary material for: Understanding Perceptions of Climate Change, Priorities, and Decision-Making among Municipalities in Lima, Peru to Better Inform Adaptation and Mitigation Planning
Source: PLoS One. 2016 Jan 25;11(1):e0147201. doi: 10.1371/journal.pone.0147201 (PMC4725729; doi:10.1371/journal.pone.0147201)
Supplement: S1 Appendix — (DOCX) [file pone.0147201.s001.docx]

**S1 Appendix**

Focus Group Questionnaire – Facilitator`s Guide

Perception and Concerns

1. In your municipality, which needs of the population are considered priorities?
   1. In your area of work?
2. What actions are performed by your municipality to implement a project?
3. What are the variables that, as a municipality, are considered to prioritize a project?
4. Do you use any sort of tool to help prioritize projects?
   1. If the answer is affirmative, which ones? Are they online, spreadsheet-based, etc.?
5. What level of priority do human health problems receive in your district?
   1. In your area of work?
6. What level of priority do environmental issues receive in your district?
   1. In your area of work?
7. Within the municipality, is there concern about climate change? Who is in charge of deciding what actions to take with respect to climate change?
   1. Government, community organizations, individuals, etc.
8. Have you received any information related to climate change from the municipality?
   1. If the answer is affirmative, what type?
9. Has your district suffered any impacts of climate change?
   1. If the answer is affirmative, could you explain?
10. What effects of climate change would be the most important in your district?
    1. Possible risks could include: water scarcity, emerging infectious diseases, hyperthermia (heat stroke), changes in precipitation patterns, sea level rise, ocean acidification.
11. How much do you think your district will be impacted by the problems associated with climate change?
    1. What are the areas that will be impacted most?
    2. Please order them according to importance to your district.
12. Are there activities in your district that contribute to climate change?
    1. Please name them.

Climate change strategies

1. Is your district currently carrying out any mitigation or adaptation actions?
   1. In your area of work?
   2. Please name them.
2. Is your district more interested in mitigation or adaptation actions?
   1. In your area of work?

Tool to evaluate adaptation/mitigation actions

1. Do you believe that learning about strategies to respond to climate change will contribute to your district and area of work?
2. Do you think that a tool (online or spreadsheet-based) could help evaluate different strategies to respond to climate change?
3. How much time could you devote to using such a tool?
   1. 30 minutes, 1 hour, 2 hours, others.
4. Would you be willing to pay for such a tool?
   1. If the answer is affirmative, how much?
